# Functions of Non-Suicidal Self-Injury Among Adolescents with Eating Disorders

**DOI:** 10.1192/bjo.2026.11083

**Published:** 2026-06-30

**Authors:** Victoria Kumekor

**Affiliations:** Birmingham and Solihull Mental Health Foundation Trust, Birmingham, United Kingdom

## Abstract

**Aims:**

Eating disorders (EDs) and non-suicidal self-injury (NSSI) frequently emerge during adolescence and often co-occur, particularly in bulimia nervosa and anorexia nervosa binge-eating/purging subtype. Although both behaviours are widely understood as maladaptive strategies for managing distress, the specific psychological functions of NSSI within ED populations remain insufficiently synthesised. This review aims to consolidate current evidence on the functions of NSSI among adolescents with EDs, drawing on theoretical frameworks such as Nock and Prinstein’s four-function model and emerging literature on affect regulation, self-punishment, interpersonal communication, and identity processes.

**Methods:**

A systematic search of PsycINFO, Embase, MEDLINE, and the Behavioural and Psychological Sciences Collection (2019–2024) was conducted using terms related to eating disorders, non-suicidal self-injury, and adolescence. Eligible studies included peer-reviewed empirical research reporting primary data on NSSI functions among adolescents with a diagnosed ED. Two reviewers independently screened studies, extracteddata, and resolved discrepancies through discussion. Extracted variables included study design, demographic characteristics, NSSI typology, and identified NSSI functions. A thematic synthesis approach was used to generate descriptive and analytical themes.

**Results:**

Eight studies met inclusion criteria, representing clinical, community, and longitudinal designs. Samples were predominantly female adolescents, with ED presentations largely characterised by binge-purge behaviours. NSSI methods included cutting, scratching, burning, hitting, and ED-specific self-endangering behaviours such as laxative misuse, insulin manipulation, and psychogenic polydipsia. Across studies, NSSI served multiple overlapping functions. The most consistent was *emotion regulation*, where self-injury was used to reduce overwhelming affect or alleviate emotional numbness. Other prominent functions included *self-punishment*, *relief from dissociation*, *interpersonal signalling of distress*, and responses to *atypical cognitions* such as quasi-psychotic experiences. Several studies also indicated that ED behaviours themselves may carry direct self-harm.

**Conclusion:**

NSSI in adolescents with EDs is multifunctional and closely intertwined with the emotional, cognitive, and interpersonal difficulties characteristic of this population. Understanding these functions is essential for developing targeted, function-focused interventions that offer safer alternatives to the needs currently met through self-injury. Further research is needed to clarify how NSSI and ED behaviours interact over time and to guide more effective treatment approaches

